# Adiponectin and Cardiovascular Risk. From Pathophysiology to Clinic: Focus on Children and Adolescents

**DOI:** 10.3390/ijms20133228

**Published:** 2019-06-30

**Authors:** Antonina Orlando, Elisa Nava, Marco Giussani, Simonetta Genovesi

**Affiliations:** 1 School of Medicine and Surgery, University of Milano-Bicocca, Milan 20100, Italy; 2 Family Pediatrician, ATS Milan 20100, Italy; 3 Department of Cardiovascular, Neural, and Metabolic Sciences, S. Luca Hospital, IRCCS, Istituto Auxologico Italiano, Milan 20100, Italy

**Keywords:** adiponectin, children, cardiovascular risk, hypertension, insulin resistance, metabolic syndrome

## Abstract

Adiponectin (Ad) is a cytokine produced by adipocytes that acts on specific receptors of several tissues through autocrine, paracrine, and endocrine signaling mechanisms. Ad is involved in the regulation of cell survival, cell growth, and apoptosis. Furthermore, Ad plays an important pathophysiological role in metabolic activities by acting on peripheral tissues involved in glucose and lipid metabolism such as skeletal muscle, and the liver. Adiponectin has anti-inflammatory, anti-atherogenic, and insulin-sensitizing effects. For this reason, low levels of Ad are associated with the development of cardiovascular complications of obesity in adulthood. Numerous studies have shown that, even in children and adolescents, Ad is associated with risk factors for cardiovascular diseases. In obese children, reduced levels of Ad have been reported and Ad plasma levels are inversely related with abdominal obesity. Moreover, lower Ad concentrations are associated with the development of metabolic syndrome, insulin resistance and hypertension in pediatric subjects. In addition to a higher prevalence of cardiovascular risk factors, plasma values of Ad are also inversely associated with early organ damage, such as an increase in carotid intima-media thickness. It has been suggested that low Ad levels in childhood might predict the development of atherosclerosis in adulthood, suggesting the possibility of using Ad to stratify cardiovascular risk in obese children. Some evidence suggests that lifestyle modification may increase Ad plasma levels. The aim of this review is to summarize the evidence on the relationship between Ad, obesity, metabolic alterations and hypertension in children and adolescents, and to address the possibility that Ad represents an early marker of cardiovascular risk in pediatric subjects. Furthermore, the effects of non-pharmacological treatment (weight loss and physical activity) on Ad levels are considered.

## 1. Introduction

The discovery, in the mid-1990s, that adipose tissue is an important endocrine organ that secretes a number of biologically active adipokines into the bloodstream was a big step forward in understanding human metabolic mechanisms. Adiponectin (Ad) is one of the main cytokines produced by adipose tissue. As Ad acts on peripheral target tissues through specific receptors, it can be classified as a hormone. To date, the metabolic effects of Ad have not yet been fully clarified. Nevertheless, numerous experimental and clinical observations clearly suggest that a decrease in Ad bioactivity is involved in the pathophysiology of cardiovascular diseases. In particular, the reduction of anti-inflammatory, antiatherogenic, and insulin-sensitizing effects of Ad increases the risk of developing cardiovascular risk factors such as as obesity, insulin-resistance, and hypertension in adults [1]. More and more evidence points to an interaction between Ad levels and cardiovascular risk playing a role from childhood onwards.

## 2. Adiponectin’s Physiology and Pathophysiology

Adiponectin was identified in 1995 as a protein secreted by adipocytes of mice and, for its homology with the complement factor C1q, was first named “adipocyte complement-related protein of 30 kDa” (ACRP30) [2]. The human Ad gene (*ADIPOQ*) was cloned by sequencing a human adipose-tissue cDNA library [3]. The gene encoding Ad spans approximately 15.8 kb and is structured in three exons on chromosome 3q27; this region has been linked to a susceptibility locus for metabolic syndrome, type two diabetes, and cardiovascular disease [4]. The full-length form of Ad is a 247 amino acid protein with four domains: an amino terminal signal sequence, a variable region, a collagenous domain, and a carboxyterminal globular domain. In the bloodstream Ad appears in different molecular weight forms, i.e., low molecular weight (trimer), medium molecular weight (hexamer), and high molecular weight (HMW, oligomer) Ad. Interestingly, these subfractions may exert different biological functions [5]. Waki et al. showed that leukocyte elastase, secreted by activated monocytes and/or neutrophils, can cleave the full-length form of Ad. This cleavage might account for the generation of the globular fragment of Ad, which is found in small proportions (1% of total Ad) in the bloodstream. The globular Ad fragment has been shown to increase insulin-stimulated glucose uptake and to boost β-oxidation of fatty acids [6,7].

It has not yet been clearly established which Ad form(s) is (are) biologically active. Based on clinical observations, the current consensus is that the HMW form is the most clinically relevant [8,9].

The major source of plasma Ad in adults are adipocytes, but recent evidence indicates that it can also be produced by other organs and tissues, such as skeletal muscle, cardiomyocytes, liver, bone marrow, and cerebrospinal fluid [10,11,12]. A certain amount of Ad was detected in human fetal tissues from 14 to 36 weeks of gestation, but the concentration declined during the progression of gestation. It was suggested that Ad may have a role during fetal development [13]. In healthy adults, plasma levels of Ad usually range from 3 to 30 μg/mL.

The mechanism of Ad secretion by adipocytes remains poorly elucidated. Adiponectin production is a multistep process that is regulated at the level of gene expression, secretion and/or formation of the multimeric forms of the protein. The molecular mechanisms involved in Ad secretion and multimerization and their regulation are yet to be fully explained. Nevertheless, fractional analyses have demonstrated that Ad is predominantly localized in the Golgi apparatus and that the secretory pathways, from Golgi to the cell surface, involve the transferrin receptor-positive endosomal system [14]. In adipocytes, the processes of Ad biosynthesis and secretion are modulated by several molecular chaperones in the endoplasmic reticulum, and after post-translational adaptations, the protein is merged into several multimeric forms. Proteolysis of the full-length protein generates the globular Ad, which subsequently acts through interaction with transmembrane G-protein-coupled receptors [15].

In 2003, two Ad receptors were identified by Kadowaky and colleagues, namely AdipoR1, a receptor for the globular form, mostly expressed in the skeletal muscle, and AdipoR2, a receptor for full-length Ad that is mainly expressed in the liver [16]. Moreover, AdipoR1 and AdipoR2 are also expressed in pancreatic cells, endothelial, smooth muscle cells, and atherosclerotic plaques. A third type of receptor (AdipoR3), specific for the HMW form of Ad, has been discovered on endothelial and smooth muscle cells [17]

T-cadherin is a membrane-associated co-receptor for Ad. It is a Glycosylphosphatidylinisotol (GPI)-anchored protein lacking intracellular domain, highly expressed in the heart, skeletal muscle and vascular tissue. T-cadherin concentrates Ad in the tissue of interest and to presents it to AdipoR1 or AdipoR2, receptors that have intracellular signaling capabilities. Consequently, it can be speculated that T-cadherin may play a role in the cardiovascular protective actions of Ad, but future studies are needed to investigate the potential signaling capabilities of this complex [18].

Several lines of evidence suggest that Ad, upon binding to its receptors, induces the activation of several downstream signaling pathways that regulate cell survival, cell growth, and apoptosis. [19]. In mice on a high-fat diet, the metabolic action of Ad is represented by stimulation of fatty-acid oxidation by muscle, thus avoiding a diet-induced rise in free-fatty acids in the plasma and causing weight loss [6]. Adiponectin can thus lead to a reduction in the tissue triglyceride content by enhancing lipid catabolism, but it can also improve insulin sensitivity, as was shown in insulin resistant mice receiving Ad [17]. The expressions of both AdipoR1 and AdipoR2 are significantly decreased in muscle and adipose tissue of insulin resistant ob/ob mice, in which reduced Ad activity resulted from a down-regulation of Ad receptors [20]. The metabolic effect of Ad in the liver is represented by a reduction of glucose production. The role of Ad in controlling hepatic glucose output is supported by studies on patients with non-alcoholic fatty liver disease, in whom an inverse relationship between plasma Ad and endogenous glucose production was described [21].

A series of in vitro and in vivo experiments demonstrated that all Ad forms act through the stimulation of the 5’-AMP-activated kinase (AMPK) in muscle, while in the liver only the HMW Ad is able to exert its biological effect [22,23,24]. The activation of AMPK by Ad influences several cellular processes in the vascular endothelial cells and the heart, leading to proangiogenic [25] and anti-apoptotic effects [26], the stimulation of nitric oxide (NO) production [27], and the reduction of myocardial infarction in a mouse model of heart ischemia–reperfusion [28]. Taken together these findings show that Ad might play a role in protection against cardiovascular diseases.

Another biological effect of Ad is mediating and enhancing the peroxisome proliferator-activated receptor-gamma (PPARγ) activities [15]. PPARγ is a nuclear receptor of ligand activated transcription factors that controls the expression of a network of genes involved in lipid metabolism, adipogenesis and inflammation. It is expressed in adipose tissue [29] and has a role in adipocyte differentiation, and in regulation of circulating levels of Ad [29]. PPARγ activation increases insulin sensitivity in different tissues (liver and skeletal muscle), and leads to release of insulin sensitizing adipokines, such as Ad and decreases inflammatory cytokines [29]. In vitro studies have demonstrated that activation of PPARγ causes an increase in Ad mRNA levels [30]; on the contrary, ablation of PPARγ gene from adipose tissue and from skeletal muscle leads to reduced circulating levels of Ad [31,32]. Moreover, PPARγ activation is able to suppress smooth muscle cell migration and proliferation and has direct vasodilatory effects. It has been demonstrated that Ad is necessary for PPARγ-mediated improvement of endothelial function in diabetic mice [33].

**Figure 1:**
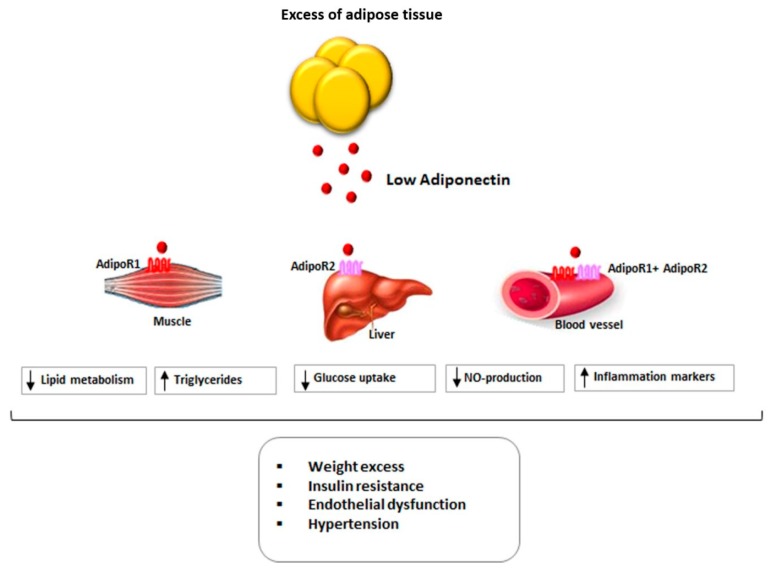
Relationship between Adiponectin levels and cardiovascular risk in obesity. Black arrow pointing up (↑): increase, Black arrow pointing down (↓): decrease.

Figure 1 summarizes the main actions of Ad in different tissues and the relationship between Ad and cardiovascular risk factors.

## 3. Adiponectin and Human Milk

Whereas Ad is primarily produced by the adipose tissue, it is also secreted by mammary glands; indeed, Ad is present in breast milk and can be absorbed by the intestine of infants [34]. A great amount of evidence demonstrates the contribution of breastfeeding in preventing excess weight and in determining a favorable epigenetic programming for the future health of the child [35,36]. It has been suggested that Ad, possibly with other cytokines present in breast milk, has a role in the mechanisms through which breastfeeding promotes proper growth and a better metabolic state [37].

The concentration of Ad is highest in colostrum and tends to decrease throughout the breastfeeding period. It is very variable and depends on different maternal factors such as parity, gestation weeks, type of delivery, exclusive breastfeeding, smoking, and ethnicity [38]. Some authors have suggested that maternal BMI may correlate with the amount of Ad in milk, but this observation has not been confirmed by other studies [39]. Regarding the correlation between Ad levels in breast milk and infant growth patterns, literature data can lead to different interpretations.

According to a recent study, breast milk Ad does not affect an infant’s body composition, while high levels of leptin and moderately high insulin values induce lower weight-by-length and BMI z-score values [40]. In developed Western countries, some authors have described an inverse correlation between breastmilk Ad and weight-for-age z-scores, weight-for-length z-scores, and body weight in the first months of life [37,41,42]. However, in the months thereafter children exposed to higher levels of Ad in milk would tend to have a greater weight for their age and length, and an increased risk of being overweight at two years of age [43,44,45]. In contrast, in developing countries, where maternal BMI values are lower, an increase in weight-for-age z-scores was observed in the tertile of children fed with milk with higher Ad concentrations, and this increase was maintained until the second year of life [46]. Breastmilk Ad may influence the growth of children in the first two years of life differently between early infancy (<6 months) and later in childhood (two years). Moreover, this effect would be conditioned by the maternal BMI value. The literature regarding the specific role of breast milk Ad, although promising, is still inconclusive. Also due to the complex interaction between breastfeeding and nutritional, metabolic, hormonal, and psycho-relational factors [40].

## 4. Adiponectin, Obesity and Metabolic Syndrome

Obesity is a multifactorial disease of epidemic proportions in industrialized countries. It results from the relationship between lifestyle factors (behaviors acquired socially or self-determined) and genetic variants (which influence the body’s response to environmental changes). The prevalence of obesity has been increasing worldwide in recent years, both in adults and in children and adolescents [47]. Becoming overweight and obesity in pediatric populations increases the risk of developing adult obesity that is associated with type two diabetes, hypertension, dyslipidemia, and metabolic syndrome [48,49,50,51]. Several studies published over the last decade reported that adipokines play an important role in glucose and lipid metabolisms, and in the development of cardiovascular and metabolic complications of obesity [52].

Adiponectin has anti-inflammatory, anti-atherogenic and insulin-sensitizing properties. Adiponectin protects against atherogenesis by modulating the cross-link between endothelial cells and platelets, by stimulating NO production, and by inhibiting the vascular endothelial growth factor (VEGF) [53].

In adults, an inverse correlation between excess weight and central obesity and Ad plasma levels has been observed [54,55], and low Ad plasma values are associated with several cardiovascular risk factors such as hypertension, insulin resistance [53,56,57,58] and metabolic syndrome [59]. Plasma levels of Ad increase after weight reduction loss in obese adult patients, suggesting that the presence of low concentrations of Ad associated with obesity is a reversible condition. Furthermore, Ad expression can be influenced by single nucleotide polymorphisms in the gene encoding this protein; so, both environmental and genetic factors can contribute to variability in the Ad concentration [60]. Reduced serum levels of Ad are also described in adult diabetic patients [61]. The down-regulation of AdipoR1/2 may play a role in the development of insulin resistance, type two diabetes, metabolic syndrome, and atherosclerosis [62].

### Evidence in Children

Reduced levels of Ad (in particular of its HMW oligomers) have been reported in obese children, compared to normal weight control subjects [63,64,65,66,67]. It has also been reported that male children with persistent obesity have lower Ad levels than those with transient obesity; therefore, low levels of Ad could be a predictor of persistent obesity among boys [68].

A negative correlation between Ad and visceral fat has been described in children and in adolescents [63,69,70]. Plasma Ad levels are inversely correlated with waist circumference, a marker of abdominal obesity [52]; moreover, weight and waist circumference-to-height ratio are independently associated with plasma Ad concentrations [64]. Other authors demonstrated that waist circumference is an independent determinant of plasma Ad concentration, suggesting that visceral adipose tissue has a central role in reducing circulating levels of Ad [67]. Moreover, high leptin to Ad ratio has been associated with BMI Z-score gain and waist to height ratio gain in non-obese children [68].

As in adults, in children and adolescents lower Ad concentrations are associated with metabolic risk factors for atherosclerosis and cardiovascular diseases (triglycerides, total cholesterol, LDL-cholesterol, fasting glucose levels, and Homeostasis model assessment (HOMA)-index) [65]. Lower levels of Ad were observed in obese adolescents with insulin resistance compared to control subjects [66]. Moreover, a negative correlation between Ad serum values and HOMA index has been shown in obese children [66]. The demonstration of an inverse relationship between plasma Ad concentration and HOMA-index suggests that Ad is directly involved in insulin resistance and in the development of diabetes [66,67]. Adiponectin mRNA levels are related to PPARγ mRNA levels in both subcutaneous tissue and omental adipose tissue in normal weight and overweight children [71]. These findings support the hypothesis of a direct relationship between Ad and PPARγ gene expression.

Obesity and insulin resistance are both involved in the development of the metabolic syndrome. The metabolic syndrome is a pathological condition characterized by hypertension, impaired fasting glucose levels, central obesity, and dyslipidemia. Its prevalence among children and adolescents is increasing worldwide. Low plasma Ad levels predict the development of metabolic syndrome in both adults [72] and children [73,74]. However, in a recent study, Nappo et al. showed that in European children, after adjustment of the multivariate regression model for BMI, Ad is no longer significantly associated with increased prevalence of metabolic syndrome, suggesting that in the pediatric population, the association of Ad with metabolic syndrome may depend on the body fat amount [75]. It has been suggested that in children and adolescents, the leptin-to-Ad ratio could be a better marker for the diagnosis of metabolic syndrome than plasma leptin or Ad levels individually and that this ratio could be a target for early cardiovascular risk prevention [74].

An association between Ad and obstructive sleep apnea (OSA) has been demonstrated in adults; in particular, Ad levels are lower in patients with OSA regardless of the concomitant presence of obesity [76]. Although OSA is common in pediatric obesity and represents an independent risk factor for metabolic syndrome, an association, independent of excess weight, between Ad and OSA, oxygen desaturation, and percentage of time of sleep in this population could not be demonstrated [77]. However, a recent study performed in a small sample of Chinese children with asthma suggests an inverse correlation between Ad levels and the severity of the disease [78].

## 5. Adiponectin and Hypertension

A systematic review and meta-analysis evaluated the epidemiological evidence of the association between plasma Ad values and arterial hypertension in adult subjects. The meta-analysis, based on 48 studies that included more than 17,000 subjects, about half of whom were hypertensive, showed that hypertensive subjects had significantly lower Ad levels than those with normal blood pressure values. Each mg/mL increase in plasma Ad was associated with a 6% reduction in hypertension prevalence [79]. Reduced levels of plasma Ad have been described in patients with metabolic syndrome and hypertension compared to subjects who are only hypertensive or suffering from metabolic syndrome without hypertension [80]. Moreover, different variants of the gene encoding Ad have been related to the concomitant presence of metabolic syndrome and hypertension, suggesting a genetic linkage between these conditions [80].

Several mechanisms are potentially implicated in the relationship between Ad and arterial hypertension. It has been suggested that reduced secretion of Ad leads to insulin resistance [81,82] and some studies have shown a relationship between plasma concentrations of Ad and the activity of the renin-angiotensin-aldosterone system [83]. Furthermore, in adulthood, low levels of Ad are associated with an increase in sympathetic activity [82]. This finding was confirmed in young girls, in whom leptin values were associated with a predominance of sympathetic activity, while Ad levels were shown to be associated with an increase in vagal tone, suggesting a more favorable profile of the autonomic balance in girls with high values of Ad [84].

### Evidence in Children

In children, the relationship between Ad and blood pressure (BP) is complex, and only partially mediated by the presence of weight excess and visceral adiposity. In a study performed in a sample of rural Chinese male adolescents, plasma Ad values were negatively correlated with those of systolic blood pressure (SBP) and this association was independent of visceral adiposity and insulin resistance, while the positive association between plasma leptin and blood pressure values disappeared after adjustment for visceral adiposity and HOMA index [85]. Brambilla and colleagues have shown that, in a population of children that included both hypertensive and normal blood pressure subjects, Ad was significantly and inversely associated with the presence of hypertension, even after adjustment for weight class, HOMA Index and presence of puberty. The same result was evident when in the statistical model the presence of a waist/height ratio >50% was entered as a predictor of hypertension instead of the presence of obesity. Moreover, the subjects with the lower plasma levels of Ad were those who had both hypertension and obesity, or both hypertension and waist/height ratio >50% at the same time. The group of children with normal weight and normal blood pressure showed the highest plasma values of Ad [64]. An important concept emerged from this study: the simultaneous presence of the two pathological conditions was associated with a more negative metabolic pattern compared to the presence of only obesity or only arterial hypertension. This observation was subsequently confirmed by Yin and colleagues who showed that in a population of obese children, the presence of arterial hypertension was associated with a significant reduction in plasma levels of Ad. Even after correction for possible confounding factors, the Ad values were inversely related to the z-score of SBP and diastolic blood pressure (DBP). In a subgroup analysis, the weight reduction led to a decrease of the blood pressure values and an increase of Ad [85]. A recent study showed that in a population of 240 Mexican children serum levels of Ad, but not those of other adipocytokines, were inversely associated with high BP values. These results were confirmed after adjustment for BMI and waist circumference [86,87]. Taken together, all these findings strongly suggest an association between low Ad levels and hypertension, which is independent of obesity.

It has been shown that in adulthood Ad levels are associated with a reduced risk of developing arterial hypertension, even after adjustment for possible confounding factors [88]. Recently a similar observation was made in a population of children and adolescents in which the risk of developing hypertension during a follow-up of six years was approximately doubled in subjects with a high leptin/adiponectin ratio. In the subgroup of subjects with metabolically healthy obesity, a high leptin/adiponectin ratio was significantly associated with an increased risk of arterial hypertension during follow-up compared to metabolically healthy normal-weight children. Furthermore, the subgroup with the greatest risk of developing arterial hypertension was the one presenting both obesity and a high leptin/adiponectin ratio [89]. Finally, it has been suggested that hypertensive children have an up-regulation of white cell Ad receptors and low Ad plasma levels compared to healthy controls [90].

## 6. Adiponectin and Organ Damage

A considerable amount of evidence shows that low Ad plasma values are associated with the presence of coronary artery disease [91]. For reasons of age, this type of evidence does not exist in the case of pediatric populations.

### Evidence in Children

In childhood, however, low plasma values of Ad seem to be associated not only with a higher prevalence of cardiovascular risk factors, but also with early organ damage. In particular, the presence of a significant inverse association between the early alterations of carotid intima-media thickness (IMT), and levels of Ad has been suggested. Beauloye et al. demonstrated, in a sample of obese children, that Ad plasma concentrations were inversely and independently related to carotid IMT, after adjustment for Tanner stage and BMI [92]. Recent data suggest a role of Ad in predicting the development of carotid atherosclerosis. In a population of Finnish adult patients, preclinical carotid atherosclerosis (presence of plaque at carotid bifurcation and/or elevated IMT) and Ad values measured in their childhood were evaluated. Ad childhood plasma levels were inversely related to the presence of preclinical carotid atherosclerosis in adulthood (OR 0.68 for 1 standard deviation increase), after adjustment for pediatric values of non-high-density cholesterol, BMI and, blood pressure. These findings support the hypothesis that Ad levels could be an emerging predictive factor in addition to traditional cardiovascular risk factors and that low Ad values may predict the development of atherosclerosis [93].

Researchers from the Bogalusa Heart Study showed that, in a population with an average follow-up of over 25 years, there was a direct correlation between BMI in childhood and IMT in adulthood, while Ad values in adulthood were inversely related to the IMT. However, these associations were evident only in subjects with plasma Ad lower than the median values of the study population. In other words, Ad played a role in mediating the relationship between excess weight in children and atherosclerosis in adulthood. These data strongly suggest the possibility of using Ad values to stratify cardiovascular risk in obese children [94].

## 7. Adiponectin and Intervention

Most of the studies on Ad in the pediatric age group focus on the relationship between this cytokine and excess weight. Pediatric obesity is a complex health problem with great costs both for the families and for society. To address the complex phenomenon of pediatric obesity, it is important to know the physiological mechanisms that regulate energy intake and metabolism. In the previous sections of the review it was widely discussed that in adults as well as in children low Ad levels are related to the presence of cardiovascular risk factors as obesity, hypertension, and diabetes. The main interventions to reduce the pathophysiological effects of obesity in childhood include an increase in physical activity and changes in the diet [95].

Dietary intervention is the starting point because obesity hormones regulate both food intake and metabolism. Nevertheless, not many studies investigated the hormonal framework in the context of weight management with dietary interventions in children and consequently data on this topic are few. A systematic review of 25 international studies in 2,153 obese children evaluated the relationships between cytokines involved in regulating energy intake and inflammation and body mass index. In 10 of 17 studies in which Ad values were measured, researchers found significant changes in pre-/post-intervention Ad levels, but the direction of the change was not univocal. Adiponectin values were increased in six studies and decreased in four studies. In six out of seven randomized trials investigators found significant increases of in Ad levels in intervention groups, compared to control groups over the duration of the study. The Authors concluded that evidence regarding the relationships between plasma Ad values and BMI modifications was inconclusive, and that this topic warrants additional research [96].

Obesity predisposes to vitamin D deficiency which affects up to 95% of patients with severe excess weight and is often associated with a low-grade inflammatory state, insulin resistance, metabolic syndrome, and type two diabetes mellitus [97]. It is debated if vitamin D administration may improve insulin sensitivity by an interaction with its modulators, such as Ad. Despite the fact that some cross-sectional studies have shown a positive association between circulating Ad and vitamin D values [98,99], meta-analytic data have failed to demonstrate a stimulatory effect of cholecalciferol supplementation on Ad levels [100]. Recently Mai and colleagues studied the effect of vitamin D on Ad, and its oligomers in non-diabetic obese subjects with vitamin D deficiency. After four weeks of follow-up, a single oral dose of 600,000 IU of cholecalciferol induced an increase of HMW Ad levels and a decrease in leptin/HMW Ad ratios, independent of changes in body weight and insulin resistance [101]. The mechanism that determines this phenomenon is not clear and further studies are needed before considering cholecalciferol supplementation a valid treatment to increase Ad in obese subjects, particularly in excess weight children.

In children physical activity is an important tool in the treatment of obesity and its complications. Moreover, exercise has an impact on the adipose tissue and the release of Ad, which is increased in order to maintain normal lipolytic activity [102]. However, inconsistent results are reported about the effect of physical activity on adipokines (leptin and Ad) in pediatric populations [103]. A recent meta-analysis including 14 randomized controlled trials (347 children) clarifies the role that exercise plays in influencing the levels of adipocytokines in obese children [104]. Adiponectin levels were assessed in 12 studies. Physical exercise led to a mean significant increase in Ad values (Weighted mean difference = 0.882 μg/mL^−1^, 95% CI, 0.271–1.493). The data also suggest that without a concomitant weight loss and change in body composition, exercise does not affect leptin levels [104]. The meta-analysis supports physical activity as therapeutic approach for obesity in children.

The main effects of non-pharmacological intervention on plasma Ad levels are summarized in Table 1.

## 8. Conclusions

Adiponectin has important anti-inflammatory, anti-atherogenic, and insulin-sensitizing properties. As in obesity plasma levels of Ad are generally low, the absence of these protective actions may explain the association of low Ad levels with the development of obesity-related cardiovascular complications in adulthood.

Numerous studies have shown that, in obese children and adolescents, reduced levels of Ad are inversely related with the amount of visceral fat. Moreover, lower Ad plasma concentrations are associated with the development of metabolic syndrome, insulin resistance, and hypertension in pediatric age populations. In addition to being related to a higher prevalence of cardiovascular risk factors, low plasma values of Ad seem to be associated also with early organ damage, such as alterations of the carotid intima-media thickness. It has been suggested that low Ad levels in childhood might predict the development of atherosclerosis in adulthood, suggesting the possibility of using Ad to stratify cardiovascular risk in obese children. In children, intervention programs based on lifestyle improvement (diet modification and an increase in physical activity) have been shown to be effective in increasing the values of Ad. Longitudinal studies designed to demonstrate that the increase in Ad levels in childhood is associated with better cardiovascular outcomes in adulthood would be very important from a clinical point of view.

## Figures and Tables

**Table 1:** Effect of non-pharmacological intervention on Adiponectin plasma levels.

Purpose	Type of intervention	Study (*n*)	Total Sample (*n*)	Results
Change in Adiponectin levels after intervention	Lifestyle (diet + physical activity) modification	9	922	four studies show that Ad levels increase after intervention
	Diet modification	3	254	one study shows that Ad levels increase after intervention
	Increase in physical activity	9	116	five studies show that Ad levels increase after intervention
	Vitamin D supplementation	7	225	two studies show that Ad levels increase after intervention

## Abbreviations

Ad	Adiponectin
AMPK	AMP-activated kinase
NO	nitric oxide
HMW	high molecular weight
AdipoR1	Ad receptor 1
AdipoR2	Ad receptor 2
AdipoR3	Ad receptor 3
GPI	Glycophosphatidylinositol
PPARγ	Peroxisome proliferator-activated receptor-gamma
BMI	Body mass index
VEGF	Vascular endothelial growth factor
HOMA	Homeostasis model assessment
OSA	Obstructive sleep apnea
SBP	Systolic blood pressure
DBP	Diastolic blood pressure
IMT	Intima-media thickness
